# Implementing quality in hospitals – stakeholders’ roles: A qualitative social network analysis

**DOI:** 10.4102/hsag.v30i0.3147

**Published:** 2025-11-14

**Authors:** Sphiwe Y. Mabena, Susan J. Armstrong

**Affiliations:** 1 Department of Nursing Education, Faculty of Health Sciences, University of the Witwatersrand, Johannesburg, South Africa

**Keywords:** compliance, key performance areas, quality standards, social network analysis, quality, tertiary hospitals

## Abstract

**Background:**

The current experience of the implementation of quality standards policy in South Africa has sometimes produced negative results because of insufficient understanding of policy goals, resource constraints, and a lack of consultation and involvement of the key actors. Understanding how the actors operate in the health establishments will assist stakeholders in improving compliance.

**Aim:**

This study aimed to examine the roles of actors responsible for the implementation of the six key performance areas of the National Core Standards (NCS) in the better- and least-performing hospitals in Gauteng.

**Setting:**

This study was conducted at two tertiary hospitals in Gauteng province.

**Methods:**

A qualitative social network analysis (SNA) using Net-Map was conducted with purposively selected staff to identify key actors, relationships and influence in implementing the NCS. Participants mapped actors using coloured nodes, arrows and influence towers. Data, including direct quotes, were collected through guided sessions and transcribed, revealing power dynamics and communication flows within two Gauteng tertiary hospitals.

**Results:**

Social network analysis revealed that one of the hospitals had a collaborative network with active chief executive officer (CEO) involvement, while another hospital followed a more hierarchical structure. Both were bounded networks with quality managers as key actors. Informal relationships enhanced coordination and compliance. Actor influence, connectivity and leadership engagement were critical in implementing the NCS, highlighting SNA’s value in diagnosing organisational strategy and performance gaps.

**Conclusion:**

Leadership support and shared interest with all other actors responsible for policy implementation are essential if the healthcare establishment is to comply with NCS.

**Contribution:**

Social network analysis can reveal bottlenecks, identify influential individuals and inform strategies for enhancing organisational effectiveness.

## Introduction

South Africa’s healthcare system is undergoing a significant transformation, with a focus on achieving universal health coverage through the implementation of the National Health Insurance (Mogakwe, Ally & Magobe 2019). The current experience of the implementation of health policy in South Africa has shown that new policies have produced unexpected and sometimes negative results because of insufficient understanding of policy goals, a lack of enforcement of existing policies, resource constraints, and absence of consultation and involvement of other actors (Muthathi & Rispel 2020). These negative outcomes include imposing barriers to access instead of removing resistance to equity promotion of the health management action and weakening the quality of care instead of enhancing the efficient use of resources (Kruk et al. 2018).

Maphumulo and Bhengu (2019) pointed out that services offered by public health institutions are not meeting the basic standards of care and patient expectations, as indicated in many media reports and audits. At first glance, this situation may not appear to be serious, as the Global Health Index (2019) ranks South Africa highest among African countries in provision of the quality healthcare services. Several studies, however, indicate that there is cause for concern, with Mogakwe (2020) referring to the health system as ‘ruined and in serious need of repair’.

In an attempt to address such issues, the Department of Health in South Africa developed a set of National Core Standards (NCS) in 2011, which were later revised to Norms and Standards, gazetted in 2018 in order to provide benchmarks for quality expectations for health establishments (National Department of Health 2018). Despite ongoing efforts by the NDoH to strengthen healthcare service delivery, numerous reports continue to highlight persistent deficiencies in quality of care provided in South Africa’s public health sector (Cape Times 2025; Hosken 2025). This indicates that the mere existence of national standards and compliance monitoring mechanisms, such as those enforced by the Office of the Health Standards Compliance (OHSC), is insufficient to drive meaningful improvements in healthcare quality (OHSC 2024; Scott, Gilson & Erasmus 2023). Effective quality improvement requires not only monitoring but also the integration of resources, leadership accountability and systemic support to address structural and operational gaps (Munyewende & Rispel 2014). The authors, however, noticed that the results of the audits carried out by the Gauteng Department of Health using the core standards audit tool demonstrated that the compliance ratings of health facilities with seemingly similar resources and mandates produced hugely varying results. As part of a larger study, the authors chose to examine the role of the actors in the hospital services in an attempt to understand how their relationships affect the quality of care delivered at the healthcare establishment.

The policy implementation framework devised by Walt and Gilson (1994), which provides a structure for analysing health policy by considering the interplay of context, content, process and actors was used to guide the larger study. The specific objective of this phase of the study, which forms the basis of this article, was to describe relationships between the actors responsible for the implementation of the six key performance areas (KPAs) in the better- and least-performing tertiary hospitals in Gauteng.

Social network analysis (SNA) was the method used to understand factors relating to compliance with the KPAs of the NCS. It is a method for studying the relationships and interactions between the individuals, groups or organisations within a network, in this case selected hospitals. In healthcare, SNA can be applied to examine how information and practices spread through professional networks, ultimately influencing the implementation of the quality standards.

### Problem statement

The majority of public health facilities in South Africa, approximately 60%, fail to meet established quality standards, with significant disparities across provinces and pervasive structural and resource-based barriers. Despite some progress in issuing compliance certificates and improving governance, persistent challenges in leadership, funding, infrastructure, staffing and security continue to undermine the quality of health service delivery (OHSC 2023/24). The NCS represent the minimum requirements for a hospital to provide for delivery of safe and cost-effective quality healthcare services, and therefore, it is of great concern that compliance is low and variable despite the different levels of hospitals ostensibly having similar resources available to them. Attempts have been made to focus attention on what are known as the six priority areas (KPAs) of the NCS; however, compliance is still poor. To date, attempts to improve compliance with the standards have been reactionary, with hospitals with low scores being criticised and their management expected to resolve issues rather than looking at the systemic issues. Analysing the operations of actors in both the better- and least-performing hospitals will help clarify the dynamics that affect compliance with the NCS. This understanding will enable stakeholders to leverage the success factors found in the top-performing hospitals and create effective strategies to improve the performance of the lowest-performing ones.

### Purpose of the study

The purpose of this study was to describe the relationships between the actors responsible for implementing the six KPAs of the NCS in the better- and least-performing hospitals in Gauteng.

## Research methods and design

### Study design

Qualitative SNA uses visual representations to demonstrate social relationships within an organisation to highlight the patterns of interconnections between actors. The method assists in uncovering complex dynamics and patterns of power and behaviour within a group of people, as well as better understanding the roles of those people. It is particularly useful to understand situations that are impacted by social, cultural, political and economic factors and provides all actors, whether often under-represented or stigmatised, an opportunity to participate in a manner that may be challenging for them through conventional methods such as surveys and interviews (Benedict et al. 2024).

A mapping process was conducted according to Net-Map 2007 and findings were analysed according to Tichy, Tushman and Fombrun (1979).

### Study setting

The study was conducted in two tertiary hospitals in the Gauteng province. The hospitals selected were found to be the ‘better’ and the ‘least’ performing hospitals based on their performance in internal quality audits held on the six KPAs of the NCS. The better- and least-performing tertiary hospitals were included in this study in order to make a meaningful comparison of the issues impacting the implementation of the NCS.

### Population and sampling

The population for this phase of the study comprised all health workers employed in the selected hospitals, as all have a direct or indirect responsibility for complying with the KPAs of the NCS. Owing to the limitation of the coronavirus disease 2019 (COVID-19) protocols that were in place at the time of data collection, staff members employed within the quality assurance departments of the selected hospitals were asked to suggest the people with whom they interact to ensure that the six KPAs are implemented. The sample for each of the SNA groups included the quality assurance manager, the complaints officer, the NCS champion and four additional staff members responsible for monitoring quality in the hospitals.

### Data collection

The data collection consisted of six steps, namely, preparation, actor selection, drawing of links, building influence towers, meeting goals and group discussion. In the preparation phase, key informants who were involved in the implementation of the six KPAs of the NCS were asked questions using the predefined questionnaire to determine which actors are involved in a given network, how they are linked to each other, how influential they are, what their goals are and how these goals are being achieved in terms of compliance with the NCS. However, because of the COVID-19 restrictions, this step had been amended and was limited to the quality assurance department staff. All other steps were carried out with the selected participants.

In step two, the participants were asked to identify who is involved in the process of implementing the KPAs in the hospital and to write their designation on cards, all of which were placed on an empty sheet known as the ‘Net-Map’ sheet. For step three, participants were asked to identify links between the actors by answering the question, ‘Who is linked to who?’ ‘Who influences who?’ Participants drew arrows between the actors. If actors exchanged something such as information, they drew a double-headed arrow. If actors exchange more than one responsibility, participants used differently coloured arrow heads to existing links. The fourth step involved representing how strongly actors influenced one another. To do this, participants built ‘towers’ of plasticine balls. It was explained to the participants that in this study, ‘influence’ referred to influence relating to the implementation of the KPAs and not influence of all the services provided at the hospital. The higher the degree of influence, the higher the tower. The influence towers were placed next to the actor cards. Step five involved a group discussion on whether each actor supported the implementation of the KPAs. This was done actor by actor. Finally, in step six, participants gave their opinions on what the network they had built meant for the strategy of the organisation – in this case, compliance with the KPAs – and what happened in the case of conflicting goals.

### Data analysis

Analysis of the data was performed according to Schiffers (2007) who cited three goals that aid the analysis of SNA data, namely: (1) visual analysis of data with participants through short feedback and allowing participants to visually and intuitively interpret the network they have drawn, (2) transforming the map into graphics for visual analysis by the researcher and (3) sharing the visuals with people who were not involved in the mapping process for discussion and verification. The completed drawings were photographed for later analysis and verification and the discussion was recorded and later transcribed.

The degree of interaction and cohesiveness of the actors were explored to identify the number of connections individual actors have based on the pictures taken during the net mapping process.

The work of Tichy et al. (1979) guided further analysis of the data, which involved determining the transactional content that examines what is exchanged by the people (e.g. information, influence or friendship; the nature of the links including the intensity, reciprocity, clarity of expectations and the multiplexity of the links; and the structural characteristics of the links). Structural characteristics include: (1) the size of the network, which influences the flow of communication, discerns information breakdown, and may create bottlenecks and structural holes as well as indicating: isolated individuals and teams; (2) network density, which refers to the number of actual links in the network that influences the frequency of information flow between individuals – a dense network is the network in which the number of ties is close to maximum; (3) openness, which refers to the number of actual external links of a social unit – in a bounded network, external links are absent; (4) reachability, which refers to the average number of links between any two individuals in the network; and (5) measures of centrality, which measures the importance of the actors within the network and shows which actors are central (Borgatti & Cross 2003). According to Tichy et al. (1979), this is determined by the position in the formal hierarchy.

### Ethical considerations

Written permission to collect data in the two selected tertiary hospitals was obtained from the Gauteng Department of Health Research Committee. Ethical clearance was obtained from the Human Research Ethics Committee (HREC) Medical of the University of the Witwatersrand (M180366). Informed consent was obtained from participants both to participate in data collection and to allow discussions to be recorded. The identity of participants in each SNA group was known within the group, but members were asked not to discuss the proceedings outside the group. The participants were identified by codes in the thesis. All data were secured – electronic data were stored in encrypted devices and protected by passwords.

## Results

The findings at each of the two hospitals will be described individually and comparisons will be drawn in the discussion section of this article.

### Findings during the mapping process at Hospital A

The participants were specific in naming individual actors by title and level of responsibility, for example, operational managers, indicating that they considered the actors’ transactions to be related to the job they were employed to do. The relationship involved interacting and communicating directly with other actors in each department in the hospital in order to implement the six KPAs of the NCS; for example, to ensure medicine availability and avoid stockouts, communication was conveyed by the head pharmacist to the chief executive officer (CEO) and all departments. To illustrate this: Participant A4 stated:

‘We work directly with our CEO and the pharmacy to make sure that stock is available and where there are stock-outs pharmacy informs the CEO and the matter is attended to urgently.’ (Participant 4, Hospital A, Female)

With regard to the nature of the links, the actors who share the closest ties are the CEO and the pharmacy manager. The CEO as a senior manager was portrayed as being hands-on in facilitating the implementation and compliance with the six KPAs of the NCS, in that he or she deals directly with the relevant department whenever there is an issue of concern. The CEO was seen to have an open-door policy, which encouraged staff members to reach out to him or her if there are matters that need his or her attention. A participant comment:

‘Since our CEO arrived in this hospital, we have seen a huge improvement on the quality standards compliance as s/he is more supportive and involved.’ (Participant 6, Hospital A, Female)

Therefore, according to the participants, there is a high level of reciprocity and intensity between other actors and the CEO, who appears to be central to facilitating links with other actors.

When reviewing the data related to the structural characteristics of Hospital A, it was seen that the actors see themselves as part of a bounded network, which is a network with a set number of network members and therefore does not have a relationship with actors outside the network (Promise 2020). This network consists of seven groups of actors comprising the hospital CEO, nursing matrons, operational managers of the hospital units, the pharmacist, a quality assurance manager, nurses and a cleaning manager. The support staff (apart from the cleaning manager) were considered to be isolated from the network that influences the implementation of six KPAs of the NCS. This was explained by participant 2, Hospital A, Female who stated that ‘frontline staff are not taken into consideration when making decisions while they are expected to comply to the standards’.

The density of the network at Hospital A revealed that although participants identified seven groupings of actors, the density varied depending on which key performance area (KPA) was required to be implemented. With regard to staff attitude, participants stated that the matron, the nurses and the quality assurance manager (QA manager) (who is also a nurse) have actual links with one another, whereas when it came to KPA implementation involving medicine supply, the QA manager, the CEO and the pharmacy manager were said to have links with one another. This reduced the density of the network but divided the network in relation to specific aspects of KPA implementation.

As Hospital A considered itself to be a bounded network, no openness with those outside the network was mentioned, but it became clear that the QA manager does, in fact, have contact with people outside the identified actors in the network.

During analysis of the mapping related to reachability in Hospital A, it was found that the QA manager interacts with all the members of the network in order to facilitate KPA implementation. Participants believe that she or he is central to the successful implementation of the KPAs and that his or her removal would result in non-compliance. The QA manager is known to staff members in all departments and can be reached by all departments, even those such as infection control who were not stated to be part of the bounded network.

With regard to centrality, the QA manager, although not the most senior person in the hospital hierarchy, was clearly identified as the key stakeholder to facilitate implementation and compliance with the quality standards as set out by the policy, and therefore the most important person within the bounded network. There was a high degree of centrality as the QA manager had direct ties with others in the network and therefore is both important and powerful. An actor’s prominence within the network reflects the extent to which they are able to influence or shape the interactions and behaviours of other actors.

In Hospital A, actors were seen to be directly linked and tied together, as all were said to be able to connect directly to each other while portraying QA manager at the centre, linked to the CEO, matron, operational managers, pharmacy and nursing. Although communication is not hierarchical in Hospital A, the QA manager has direct communication to all actors in the network in facilitating the implementation of the six KPAs of the NCS. Participant 6, Hospital A, Female affirmed that, ‘Relationship of QA manager and other departments is good as we do interact on a daily basis with wards and pharmacy communicates if there are matters that affect pharmacy’.

**FIGURE 1:**
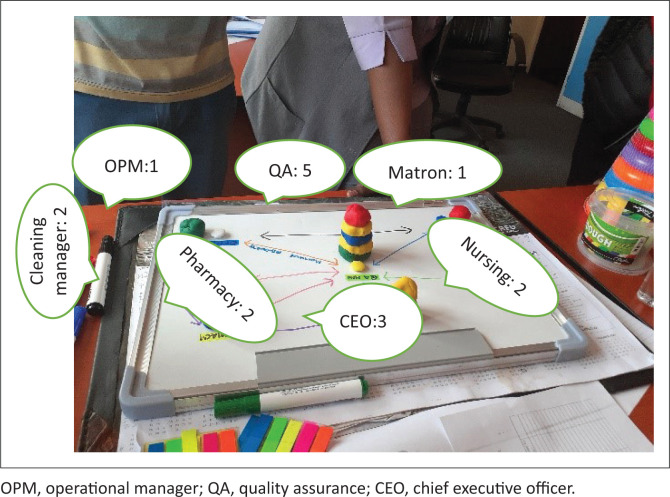
Hospital A – Tower of influence.

The relative influence or power of the QA manager is illustrated in Figure 1, which was built by participants in Hospital A.

### Findings during the mapping process at Hospital B

The participants in Hospital B also saw transactional content in terms of their assigned duties, which included exchanging information but did not specifically mention influence, although this was an element that became clear during discussion.

With regard to the nature of the actor’s links, Hospital B grouped the actors according to the department rather than individual key functions. A participant said:

‘The CEO, Quality manager, Infection control coordinator, Executive Committee [*EXCO*] as a whole, cleaning department and operational managers as the champions of quality in their units are the main drivers to make sure that implementation and compliance on the NCS happens in this hospital.’ (Participant 1, Hospital B, Female)

Thus, in Hospital B, implementation of the quality standards was monitored by the senior team, who drive performance by the lower-order staff, with the senior staff playing a more supervisory role. Furthermore, in Hospital B, quality standards such as staff attitude and availability of medicines were not mentioned individually as was done in Hospital A.

Structural characteristics in Hospital B showed that six groupings of actors who were part of a bounded network and who influence the implementation of the KPAs were identified by participants. Some ambivalence was observed regarding the composition and functioning of the hospital’s executive committee (EXCO), which comprised the chief executive officer, departmental managers, the quality assurance manager, assistant directors of nursing, operational managers, and support staff from various units. To facilitate the process, the EXCO was broken down into its six component parts. As most of Hospital B had involved frontline staff as they were represented on EXCO, their actual role appeared to be limited in terms of accountability of individuals, thus creating a potential for structural holes and bottlenecks.

Hospital B, participant 1, female said, ‘We interact with the CEO office rather than directly with the CEO and meet our CEO during EXCO meetings’. So, in Hospital B, as there is more interaction and connection with the CEO, the interaction is only done directly once a month during formal monthly meetings.

The density of Hospital B’s network connections showed that the QA manager is connected to the assistant director (AD) of nursing and the operational manager. Further analysis of network density of the senior management showed that the QA manager is connected between the CEO and the EXCO.

As with Hospital A, the participants in Hospital B considered themselves to be a bounded network, with no openness to those outside the network. There was some indication that the executive committee as an entity was seen as a separate bounded network, which would not facilitate interaction.

In Hospital B, reachability was directly related to the strength of relationships displayed between the QA manager and both the CEO and between the CEO and the EXCO, as all these actors share the shortest path that signify strong ties and relations in implementing the six KPAs of the NCS. However, the QA manager shared the longest path with the EXCO, which indicates a weak tie and relation.

Participants in Hospital B stated that communication occurs in hierarchical order to reach out to all the departments or units. In Hospital B, the nodes were primarily connected indirectly through hierarchical channels, with relationships shaped by organisational levels, although some direct communication between nodes across different levels was also evident. It was also found in Hospital B that the quality assurance department has a direct communication with all the actors. One participant stated:

‘Quality assurance department is the key to unlock implementation of the standards in this hospital as we are directly communicating to support staff if there is a complaint or non-compliance.’ (Participant 1, Hospital B, Female)

**FIGURE 2:**
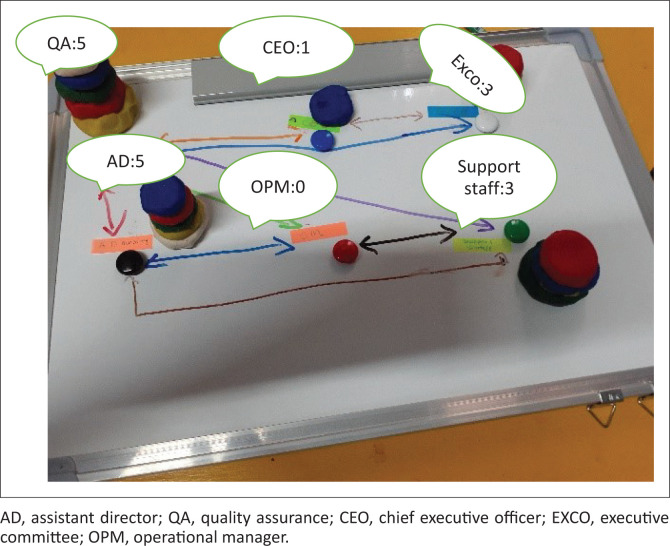
Hospital B – Tower of influence.

When examining centrality in Hospital B, the QA manager has high influence in implementing the KPAs with five levels of tower together with the assistant director (AD) nursing (matrons) who had the same degree of influence. The CEO in Hospital A was less influential, as he or she was given only one level, as opposed to the support staff who have three levels of influence in the tower (Figure 2). In Hospital B, the CEO interacts with the QA department during EXCO meetings that are held monthly. This indicates that the CEO has less power or influence, as there might be delays in communicating matters that affect the implementation of the NCS.

In addition, Hospital B operational managers are displayed as not being influential at all in implementing the quality standards, so this becomes contradictory, as operational managers in Hospital B were said to be the champions in implementing the NCS in their own units. This denotes that operational managers were given the responsibility and accountability to be the champions of quality standards but did not fulfil this role, which led to non-compliance and poor implementation of such policy.

### Measures of trustworthiness

Trustworthiness in the study was maintained and ensured through the four criteria of credibility, dependability, confirmability and transferability, as established by Lincoln and Guba’s framework in Shenton (2004:64), as applied in Jones (2021).

Credibility was assisted by recording and transcribing all discussions that took place during the SNA sessions and photographs were taken during the mapping process. In addition, an audit trail of records collected during the study was kept. To ensure dependability and therefore to assist a separate researcher in revealing similar findings, the methodology used was written up extensively in the main study, as qualitative SNA is not a well-known method of research. This requires the researcher to take an objective reading of what they have seen and heard during the research, putting aside all prior expectations, prejudices and stereotypes that could warp their interpretation (Quantilope 2024). Confirmability, as stated in Shenton (2004:72), refers to the data being an accurate reflection of the information supplied by the participants with nothing added by the researcher. The mapping was photographed, and the discussions were recorded and confirmed by the first author’s supervisor. The findings can be confirmed by an independent person. This can be determined by the recorded audio from the interviews where the voices of the participants can be heard (Jones 2021). Because of the limitations of the study, the authors are not certain that the results are transferable, but as a comprehensive description of the study was provided, this should aid transferability.

## Discussion

As the purpose of this phase of the study was to understand how the relationships between various actors in the hospitals impact the quality of care delivered at the healthcare establishment, a comparison was made between the ‘better’-performing hospital (Hospital A) and the ‘least’-performing hospital (Hospital B). As they were both tertiary hospitals in the same province, the authors assumed that access to resources was similar and yet there were considerable differences in the two hospitals’ compliance with the KPAs.

Both hospitals defined the actors in terms of their professional roles or job descriptions. The transactional nature of the relationship between actors was mainly based on the exchange of information in both hospitals. The nature of the links between actors, however, differed significantly. In Hospital A, there was a high level of reciprocity, whereas in Hospital B, relationships were hierarchical and seen as being confined to their roles as members of the various departments rather than their broader key functions within the organisation.

In Hospital A, despite the participants’ contention that the CEO maintained an open-door policy enabling all actors to communicate with him or her, in reality, the links between the CEO and other actors were weak and confined to their specific role – for example, between the pharmacists and the CEO who communicated specifically about medication management rather than broader issues. Hospital B’s CEO appeared to remain in a relative ‘bubble’, communicating with members of the executive committee but not seemingly making much attempt to influence other members of the hospital team and presumably relying on the hierarchical arrangements to communicate messages to groupings of staff members.

The role of the QA manager in both hospitals was seen to be more important in ensuring the KPAs were implemented than that of other members of the management team. In Hospital A, the CEO was seen to have an important function followed by the pharmacy manager, the nurses and the cleaning manager. The operational managers and the assistant director (matron) were seen to be minor role players with little influence in the implementation of the KPAs. The support staff were not even seen to be actors with respect to the implementation of the KPAs.

By contrast, in Hospital B, the assistant director (matron) was seen to be as influential as the QA manager, but the CEO was seen to have a minor role. It is important, however, to observe that in Hospital B, the executive team as an entity was seen to have an important role. The support staff were seen to have a role as important as the executive team, whereas the operational managers were not seen to have an important role or be influential.

Collaboration among health workers is known to improve quality of care (McLaney et al. 2022), and the role of management in quality improvement is also recognised (Bhati, Deogade & Kanyal 2023). However, the role of the quality assurance officer, while being described in many webpages, does not appear to have been well researched. An article by Rondeau and Birdi (2005) described the role extensively.

**FIGURE 3:**
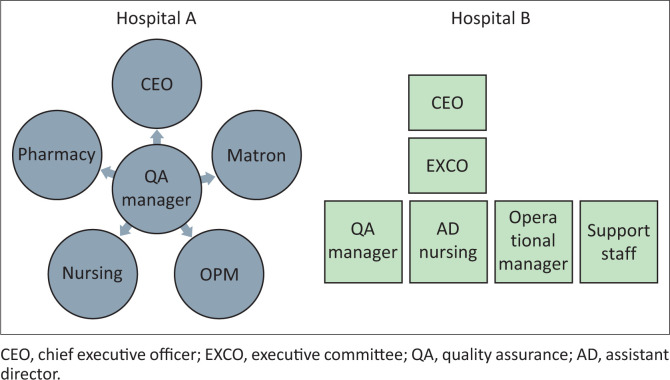
Comparison of network measure of centrality characteristics between Hospital A and Hospital B.

The influence of the quality assurance manager in both hospitals was seen to be greater than that of other actors in implementing the KPAs. What stood out from comparing the data of the two hospitals was that in Hospital A, the quality manager was central to the role, interacting with everyone in the team and having direct access to the CEO. In Hospital B, he or she only related to the CEO as one of the members of the EXCO and appeared to have a close relationship with the nurses compared to other actors. This becomes clear when examining the diagram constructed from the network measure of centrality characteristics, as shown in Figure 3.

In examining the diagrams emanating from the study and listening to the recordings of the discussions that took place during the exercise at each of the two hospitals, it is evident that there are internal challenges in both hospitals, which negatively influence the successful implementation of the KPAs.

A lack of role clarification and expectations leads to poor implementation, which may jeopardise compliance. Although there was evidence in Hospital A that actors had a clearer understanding of their key functions related to compliance with the KPAs, Hospital B actors are not assigned specific activities that aid implementation and compliance with the quality standards, with the exception of the quality manager who cannot take sole accountability for implementation of the KPAs. Zungu (2024) points out that role clarity and expectations are fundamental drivers of accountability. A lack of role clarification will inevitably result in a lack of accountability.

A lack of involvement and support from all stakeholders was evident in both hospitals. In Hospital A, certain aspects such as cleanliness and medicine supply were led by the active involvement of the CEO, whereas other aspects were left to the QA manager and other staff member while remaining informed of activities. In Hospital B, the CEO had a ‘hands-off’ approach, and actors saw quality audits as yet another hurdle to overcome rather than as a means to improve quality for patients and increase the odds of receiving a good compliance report. The concern regarding top management neglecting to show a high level of commitment to policy implementation is that staff members, or actors, are negatively affected and are less likely to adhere to organisational goals and procedures (Bashar et al. 2024).

While reviewing the size of the network in both hospitals, although the size at both hospitals was said to be similar, in Hospital B, it was a much more complex arrangement, with the EXCO being cited as one set of actors, and several other aspects related to structural characteristics differed. In Hospital A, the support staff was isolated from the network that influences the implementation of the KPAs. In Hospital B, although the frontline staff were ostensibly involved, fusion of actors in the EXCO could create structural holes and bottlenecks. This can lead to a break in the flow of communication and created information breakdown. According to Irawan et al. (2024), effective communication is essential for policy implementation.

An actor in an organisation may become a strategic actor because of formal role assigned to the individual, as in the case of quality manager. Denis et al. (2009, cited in Ludvig & Stenberg 2012) identified a ‘sense-maker in chief’ as someone tasked with shaping the strategic change and conceptually influencing how meaning is made concerning organisational change. Balogun et al. (2005, cited in Ludvig & Stenberg 2012) further presents the ‘boundary shaker’ as an individual tasked with the implementation of change across existing organisational boundaries. Moreover, an actor may also influence strategic issues because of his or her personal characteristics and competencies.

The SNA has been used to analyse the role of actors in supporting the implementation of the six KPAs of the NCS. The QA managers were seen as the key actors in supporting the organisational goal of compliance and implementation of the quality standards in both hospitals. During the mapping, it has been found that QA managers in both Hospitals A and B were portrayed as the key actors who are able to interact with all other actors. Thus, at Hospital B, it was further stated that during walkabouts, when the quality marshals observe an area that needs cleaning services within the hospital and when there is non-compliance in the cleaning services, communication occurs directly from QA manager to the cleaning manager, who then sends the team to clean the identified area. The cleaning manager at Hospital B was identified as the one who should make available cleaning materials and supervise the overall cleaning services of the hospital working together with the operational managers.

Interligi (2010) argues that compliance is a critical management function that attracts significant financial resources in supporting the organisational goal. Therefore, management can make it difficult for the organisation to close compliance gaps in quality standards if it is not directly involved in addressing such gaps. Policy provides the legal mandate to promote standardisation of quality standards and their implementation for compliance. Thus, compliance with quality standards is a strategic pillar of organisation leadership. Results of this study support the notion that a single actor cannot support organisational goals, but rather a collaborative effort is needed to make the organisational goal more achievable.

Hartley, Benington and Binns (1997, cited in Ludvig & Stenberg 2012) assert that actors who attempt to influence other actors in the public organisations face particular challenges because of the multiple and sometimes conflicting agendas and diffuse power bases among different actors. Barnett and Duvall (2005) define power as the production in and through social relations of effects that shape the capacities of actors to determine their circumstances and fate. Power dynamics influence the implementation and compliance of the six KPAs of the NCS and determine how actors interact with each other, further influencing the outcomes of interactions – in this case, compliance.

Failure of leadership can highly compromise organisational strategy in meeting policy and legal mandates. Thus, relationship and interaction of actors within the network are critical for organisational strategy. The SNA can assist in diagnosing mishaps between the actors of the network. Given the strategic importance of the decisions that the management makes, it provides insight into ways to improve effectiveness for organisational strategy and improve network connectivity.

The processes that have assisted implementation were open and good communication, involvement and support of leadership, and involvement of all stakeholders; however, these enablers were only observed in a few sections within the hospitals. The authors were left with the impression that the quality managers and many of the other stakeholders (the actors) interviewed and included in the SNA were keen to comply but felt overwhelmed by the task, which was not shared equitably among actors. This may result in compliance for its own sake, where actors engage only when an inspection is imminent, rather than collaborating continuously to maintain the established standards.

### Strengths and limitations

Qualitative social networking analysis provides valuable insights; however, it is not widely used, making it difficult to compare procedures with previous studies and to learn from prior experiences. It is possible that social desirability bias crept into the social networking analysis, as the SNA exercise was conducted in the presence of quality managers. Other participants may not have felt free to refute what was said by the quality managers. The SNA primarily describes relationships and does not necessarily establish causal links. As this was a comparative study between the better- and least-performing hospitals, only those two establishments were included in the study. Results from other levels of hospitals and other tertiary hospitals with different leaders may well vary. The results, however, have produced some unique insights into how relationships within a hospital may impact the implementation of KPAs and quality assurance in general.

### Recommendations

To improve compliance with quality standards, a culture of open communication among all role players needs to be established. Leadership support and involvement should be prioritised to create an enabling environment for effective policy implementation. Social network analysis could be more widely utilised in quality improvement studies, as it highlights the critical role of relationships and stakeholder interactions in policy implementation, offering a deeper understanding of challenges within the healthcare sector. In addition, longitudinal studies that examine how networks evolve throughout the implementation process would be valuable. Combining SNA with other methodologies, such as qualitative approaches, would provide a more comprehensive understanding of the dynamics involved. There is also a need to develop standardised SNA tools and metrics specifically tailored to healthcare quality improvement initiatives. Finally, exploring the use of SNA to predict the success of quality standards implementation could offer valuable predictive insights to strengthen future interventions.

## Conclusion

Quality of healthcare in South Africa is indeed at a crossroads, faced with the need for substantial reforms to address longstanding issues. To balance the needs of a diverse population while striving for equitable access to high-quality care requires collaborative efforts among government, healthcare providers and communities. Social network analysis offers a promising approach to understanding and improving the implementation of healthcare quality standards. It further provides insight into the relationships and interactions within the healthcare systems, helping the organisations to optimise quality improvement efforts. It is clear from this study that policy in itself can neither resolve the problems in the health services nor improve the quality of care unless the many issues hampering its implementation are addressed.

## References

[CIT0001] Balogun, J., Gleadle, P., Hailey, V.H. & Willmott, H., 2005, ‘Managing change across boundaries: Boundary-shaking practices’, *British Journal of Management* 16(4), 261–278. 10.1111/j.1467-8551.2005.00463.x

[CIT0002] Barnett, M. & Duvall, R., 2005, ‘Power in international politics’, *International Organization* 59(1), 39–75. 10.1017/S0020818305050010

[CIT0003] Bashar, A., Wasiq, M., Nyagadza, B. & Maziriri, E., 2024, ‘Emerging trends in social media marketing: A retrospective review using data mining and bibliometric analysis’, *Future Business Journal* 10(1), 1–26. 10.1186/s43093-024-00308-6

[CIT0004] Benedict, B., Lee, S., Jarvis, C., Siebeneck, L. & Wolfe, R., 2023, ‘Utilizing qualitative data for social network analysis in disaster research: Opportunities, challenges, and an illustration’, *Disasters* 48, 1–15. 10.1111/disa.12605 37471176

[CIT0005] Bhati, D., Deogade, M.S. & Kanyal, D., 2023, ‘Improving patient outcomes through effective hospital administration: A comprehensive review’, *Cureus* 15(10), e47731. 10.7759/cureus.47731 38021686 PMC10676194

[CIT0006] Borgatti, S.P. & Cross, R., 2003, ‘A relational view of information seeking and learning in social networks’, *Management Science* 49(4), 432–445. 10.1287/mnsc.49.4.432.14428

[CIT0007] Cape Times, 2025, *Nearly 2,000 clinics, hospitals found non-compliant, IOL*, viewed 27 August 2025, from https://www.iol.co.za/capetimes/news/nearly-2-000-clinics-hospitals-found-non-compliant-1418cc0c-7715-4e0f-b234-a4888aa84c54.

[CIT0008] Denis, J.L., Lamothe, L., Langley, A., Breton, M., Gervais, J., Trottier, L.H. et al., 2009, ‘The reciprocal dynamics of organizing and sensettier, L.Her, L.Halementation of major public-sector reforms’, *Canadian Public Administration* 52(2), 225–248.

[CIT0009] Digital Promise, 2020, *SNA toolkit*, viewed 26 June 2020, from https://digitalpromise.org/wp-content/uploads/2018/09/SNA-Toolkit.pdf.

[CIT0010] Global Health Security (GHS) Index, 2019, *Johns Hopkins Center for Health Security and Economist Intelligence Unit*, Nuclear Threat Initiative, viewed 28 October 2025, from https://ghsindex.org/wp-content/uploads/2019/10/2019-Global-Health-Security-Index.pdf.

[CIT0011] Hartley, J., Benington, J. & Binns, P., 1997, ‘Researching the roles of internal-change agents in the management of organizational change’, *British Journal of Management* 8(1), 61–73. 10.1111/1467-8551.00040

[CIT0012] Hosken, G., 2025, ‘Unsafe and substandard: Is that what public healthcare in SA looks like?’, *Sunday Times Daily via TimesLIVE*, viewed 27 August 2025, from https://www.timeslive.co.za/sunday-times-daily/news/2025-08-15-unsafe-and-substandard-is-that-what-public-health-care-in-sa-looks-like/.

[CIT0013] Interligi, L., 2010, ‘Compliance culture: A conceptual framework’, *Journal of Management & Organization* 16(2), 235–249. 10.5172/jmo.16.2.235

[CIT0014] Irawan, B., Roesminingsih, M.V., Widodo, B.S. & Roesminingsih, E., 2024, ‘The influence of communication on policy implementation: The mediating role of disposition’, *Decision Science Letters* 13(1), 19–28. 10.5267/j.dsl.2023.12.005

[CIT0015] Jones, J., 2021, ‘Experiences of professional nurses in providing support to student nurses in the clinical practice environment of a private hospital in Gauteng’, MCur thesis, University of the Witwatersrand.

[CIT0016] Kruk, M.E., Gage, A.D., Arsenault, C., Jordan, K., Leslie, H.H., Roder-DeWan, S. et al., 2018, ‘High-quality health systems in the Sustainable Development Goals era: Time for a revolution’, *The Lancet Global Health* 6(11), e1196–e1252. 30196093 10.1016/S2214-109X(18)30386-3 PMC7734391

[CIT0017] Ludvig, K. & Stenberg, A., 2012, ‘The actors and their roles in the meaning making process of an energy target’, in S.D. Smith (ed.), Procs 28th Annual ARCOM Conference, Association of Researchers in Construction Management, Edinburgh, UK, September 3–5, 2012, pp. 851–861.

[CIT0018] Maphumulo, W.T. & Bhengu, B.R., 2019, ‘Challenges of quality improvement in the healthcare of South Africa post-apartheid: A critical review’, *Curationis* 42(1), 1–9. 10.4102/curationis.v42i1.1901 PMC6556866 31170800

[CIT0019] Matsoso, P., 2011, *National core standards for health establishments in South Africa*, Tshwane.

[CIT0020] McLaney, E., Morassaei, S., Hughes, L., Davies, R., Campbell, M. & Di Prospero, L., 2022, ‘A framework for interprofessional team collaboration in a hospital setting: Advancing team competencies and behaviours’, *Healthcare Management Forum* 35(2), 112–117. 10.1177/08404704211063584 35057649 PMC8873279

[CIT0021] Mogakwe, L., 2020, ‘Reasons for non-compliance with quality standards at primary healthcare clinics in Ekurhuleni, South Africa’, *African Journal of Primary Health Care & Family Medicine* 12(1), 1–9. 10.4102/phcfm.v12i1.2179 PMC7284153 32501028

[CIT0022] Mogakwe, L.J., Ally, H. & Magobe, N.B.D., 2019, ‘Recommendations to facilitate managers’ compliance with quality standards at primary health care clinics’, *Curationis* 42(1), e1–e8. 10.4102/curationis.v42i1.1984 PMC6489143 31038329

[CIT0023] Munyewende, P.O. & Rispel, L.C., 2014, ‘Nurses’ perceptions of the implementation of performance management and development system (PMDS) in primary healthcare settings in South Africa’, *Global Health Action* 7(1), 25715. 10.3402/gha.v7.25715

[CIT0024] Muthathi, I.S. & Rispel, L.C., 2020, ‘Policy context, coherence and disjuncture in the implementation of the ideal clinic realisation and maintenance programme in the Gauteng and Mpumalanga provinces of South Africa’, *Health Research Policy and Systems* 18(1), 1–15. 10.1186/s12961-020-00567-z 32493349 PMC7268221

[CIT0025] National Department of Health, 2018, *Norms and standards regulation applicable to different categories of health establishments*, Government Gazette, Pretoria.

[CIT0026] Office of the Health Standards Compliance (OHSC), 2024, *Annual performance report*, Pretoria, viewed 27 August 2025, from https://ohsc.org.za/highlights-office-of-health-standards-compliance-2023-24-annual-performance-report/.

[CIT0027] Quantilope, 2024, ‘Dependability and trustworthiness in qualitative research’, *Quantilope Blog*, 29 January, viewed 10 September 2025, from https://www.quantilope.com/resources/glossary-trustworthiness-in-qualitative-research.

[CIT0028] Rondeau, K.V. & Birdi, N., 2005, ‘The role and function of quality assurance officers in Ontario hospitals’, *Quality Assurance Journal* 9(3), 179–185. 10.1002/qaj.335

[CIT0029] Schiffer, E., 2007, ‘Net-map – Influence mapping of social networks: Manual’, presented at the Sunbelt Conference of the International Network of Social Network Analysis, 01–06 May, Corfu.

[CIT0030] Scott, V., Gilson, L. & Erasmus, E., 2023, ‘Resilient governance of health systems: Focusing on resilience as a governance challenge’, *Health Policy and Planning* 38(2), 135–145. 10.1093/heapol/czad002

[CIT0031] Shenton, A., 2004, ‘Strategies for ensuring trustworthiness in qualitative research projects’, *Education for Information* 22(2), 63–75. 10.3233/EFI-2004-22201

[CIT0032] Tichy, N.M., Tushman, M.L. & Fombrun, C., 1979, ‘Social network analysis for organizations’, *Academy of Management Review* 4(4), 507–519. 10.2307/257851

[CIT0033] Walt, G. & Gilson, L., 1994, ‘Reforming the health sector in developing countries: The central role of policy analysis’, *Health Policy and Planning* 9(4), 353–370. 10.1093/heapol/9.4.353 10139469

[CIT0034] Zungu, N., 2024, *The perceived factors that drive accountability in private sector organisations of South Africa*, University of Pretoria, Pretoria.

